# 3D-QSAR reloaded: Open3DALIGN meets COSMOsar3D

**DOI:** 10.1186/1758-2946-5-S1-O13

**Published:** 2013-03-22

**Authors:** Paolo Tosco, Andreas Klamt

**Affiliations:** 1 Department of Drug Science and Technology, University of Turin, Torino, 10125, Italy; 2 COSMOlogic GmbH and Co. KG, Leverkusen, 51381, Germany

## 

A novel set of 3D descriptors, based on local grid-based COSMO σ-profiles (LSPs), has been recently proposed as a promising alternative to force-field based MIFs in 3D-QSAR [1]. These descriptors are grounded in the quantum chemistry-based COSMO-RS theory, which has become one of the methods of choice for the prediction of fluid phase equilibrium constants (e.g., partition coefficients, solubilities, vapor pressures) in pharmaceutical chemistry and chemical engineering.

**Figure 1:**
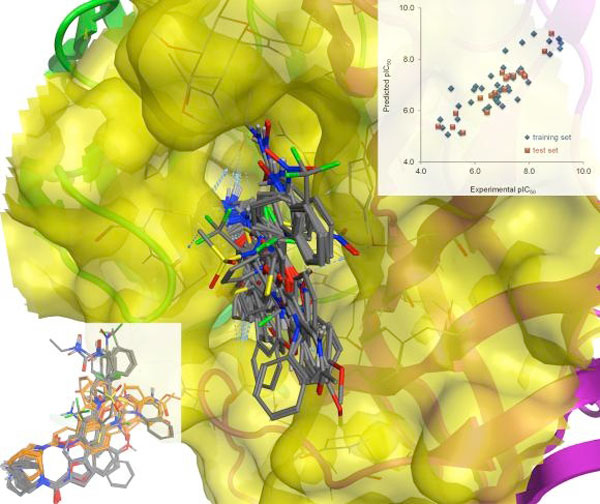
***α_7 _*ligand binding mode hypothesis (left); experimental vs predicted pIC50 plot (top)**.

Herein we present two applications of COSMOsar3D to ligand-based and structure-based modeling. In the first case, we formulated a binding mode hypothesis for a series of 59 partial agonists at the human *α_7 _*nicotinic receptor using an iterative unsupervised alignment procedure implemented in Open3DALIGN [2]. Our hypothesis is compatible with site-directed mutagenesis data and fits an independent *α_7 _*receptor homology model; the associated COSMOsar3D model has excellent predictive power (Figure 1).

**Figure 2:**
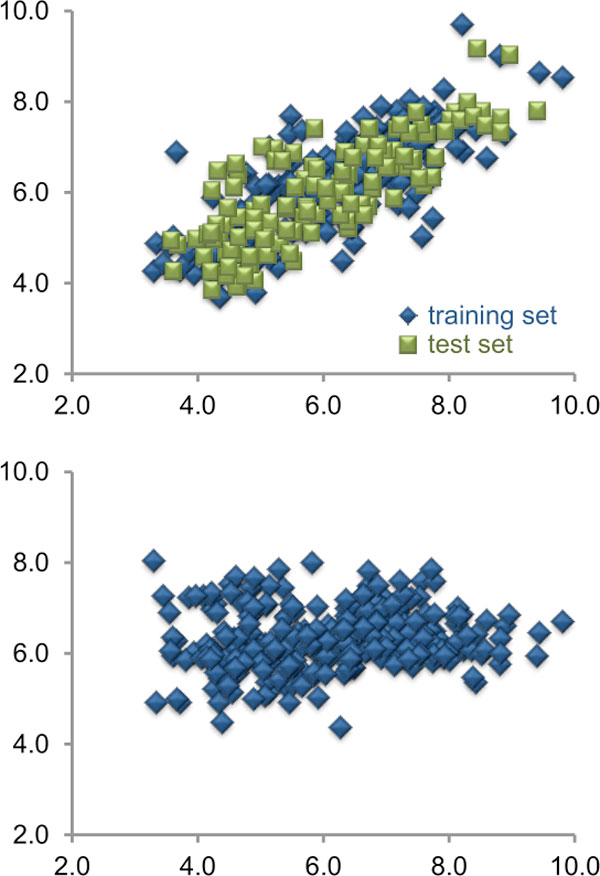
**Comparison between the accuracy of pIC50 predictions made by COSMOsar3D (top) and AutoDock VINA (bottom)**.

In the second case we used the Sutherland datasets to compare the accuracy of pIC50 predictions based on AutoDock VINA scores to those based on COSMOsar3D models Open3DALIGN'ed on co-crystallized templates; in this context, COSMOsar3D predictions proved to be far more effective in ranking ligand potencies (Figure 2).
